# Probiotics in Functional Dyspepsia

**DOI:** 10.3390/microorganisms11020351

**Published:** 2023-01-31

**Authors:** Georgios Tziatzios, Paraskevas Gkolfakis, Gabriela Leite, Ruchi Mathur, Georgia Damoraki, Evangelos J. Giamarellos-Bourboulis, Konstantinos Triantafyllou

**Affiliations:** 1 Department of Gastroenterology, “Konstantopoulio-Patision” General Hospital, 3–5, Theodorou Konstantopoulou Street, Nea Ionia, 142 33 Athens, Greece; 2 Medically Associated Science and Technology (MAST) Program, Cedars-Sinai, Los Angeles, CA 90048, USA; 3 4th Department of Internal Medicine, Medical School, National and Kapodistrian University of Athens, 157 72 Athens, Greece; 4 Hepatogastroenterology Unit, Second Department of Internal Medicine—Propaedeutic, Medical School, Research Institute and Diabetes Center, “Attikon” University General Hospital, National and Kapodistrian University of Athens, 124 62 Athens, Greece

**Keywords:** probiotics, functional, dyspepsia, treatment

## Abstract

Functional dyspepsia (FD) is a common disorder in everyday clinical practice identified nowadays as a multi-factorial, difficult to treat condition with a significant burden on patients’ quality of life (QoL) and healthcare systems worldwide. Despite its high prevalence in the general population, the precise etiology of the disorder remains elusive, with its pathophysiological spectrum evolving over time, including variable potential mechanisms, i.e., impaired gastric accommodation, gastric motor disorders, hypersensitivity to gastric distention, disorders of the brain–gut axis, as well as less evident ones, i.e., altered duodenal microbiota composition and genetic susceptibility. In light of these implications, a definitive, universal treatment that could be beneficial for all FD patients is not available yet. Recently, probiotics have been suggested to be an effective therapeutic option that could alleviate gastrointestinal symptoms in patients with Irritable Bowel Syndrome (IBS), potentially due to anti-inflammatory properties or by modulating the complex bidirectional interactions between gastrointestinal microbiota and host crosstalk; however, their impact on the multiple aspects of FD remains ambiguous. In this review, we aim to summarize all currently available evidence for the efficacy of probiotics as a novel therapeutic approach for FD.

## 1. Introduction

Functional dyspepsia (FD) is a chronic and remitting disorder originating from the gastroduodenal region, presenting as epigastric pain or burning, postprandial fullness, or early satiation with no evidence of other disease on routine investigations, including a mandatory upper gastrointestinal (GI) endoscopy to establish a firm diagnosis [1]. The current standard for the diagnosis of FD is the Rome IV criteria, which further subclassify two distinct FD subtypes, namely the postprandial distress syndrome (PDS) and epigastric pain syndrome (EPS) [2]. As far as the pathophysiology of the disease is considered, the Rome IV criteria, as well as a recent multinational consensus of European experts, support the role for impaired gastric accommodation, gastric distention hypersensitivity, disturbances in gastric emptying, and altered central nervous system signals processing [3]. Still, there are several lines of evidence suggesting that both loco regional duodenal and systemic changes may also be present in FD. Duodenal eosinophilia, epithelial barrier defect, and subtle mucosal inflammation, along with higher levels of mast cells, have been reported in FD, whereas the role of local and systemic inflammatory changes and increased small bowel-homing T cells were not until very recently highlighted [4]. These uncertainties in terms of the underlying pathophysiology limit our therapeutic options, constituting the overall treatment of these patients problematic. To date, only acid suppression therapy with proton-pump inhibitors has been shown to be beneficial for FD [3,5]; however, the results regarding the long-term efficacy even for this established treatment are lacking, while it should be noted that a considerable amount of patients still fail to respond [6]. In this context, targeting duodenal microbiota dysbiosis, as an effort to alleviate symptoms, could be a valuable alternative [7,8]. Probiotics are live microorganisms that contribute an important health benefit on the host and have been shown to be beneficial on to onset and symptoms progression of patients with IBS, presumably due to the anti-inflammatory effect, or by visceral hypersensitivity modulation [9]. Similar to IBS, accumulating evidence supports the notion that treatment with these agents could be an evolving concept also in FD by restoring GI flora alterations [10]. The aim of this review is to summarize the latest evidence available for the use of probiotics in FD.

## 2. Materials and Methods

A computerized search for studies published in the PubMed electronic database in the English language up to November 2022 was conducted using the following key words: “functional dyspepsia” as medical subject headings (MeSH) and as free-text terms combined with the Boolean set operator “AND” with the term “probiotics” as a medical subject heading and as a free-text term. The full strategy is available in Supplementary Table S1. The search was performed on 14 November 2022. After duplicate removal, titles and abstracts of all results were assessed for inclusion, while references of all eligible studies were searched manually to identify potential studies missed during the first search. Any type of trial published as full text in the English language was considered eligible for inclusion, while non-human or ex vivo studies, pediatric studies, editorials, narrative reviews, conference abstracts, studies that did not detail patient information, and case reports/series were excluded. Ultimately, 8 articles were selected for this narrative review; all evidence is presented in a top-down approach aiming to underline the context of each study.

## 3. Probiotics as a Potential Treatment for FD

**Figure 1:**
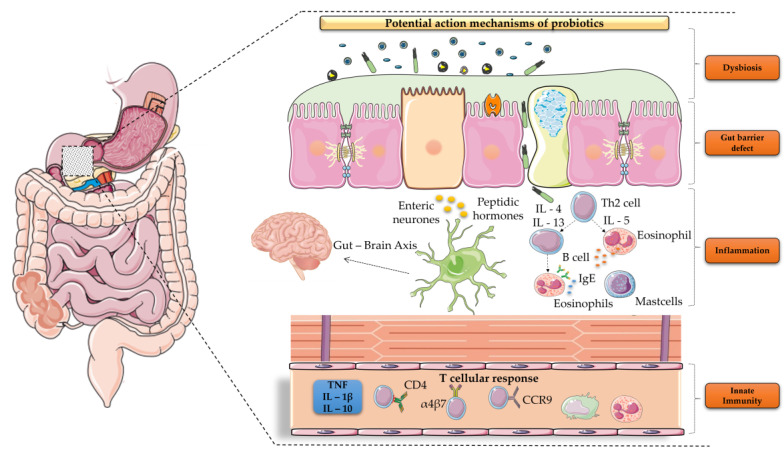
Putative mechanisms of probiotics involvement in FD treatment.

Our knowledge about the mechanisms of probiotics implications on the amelioration of FD symptoms remains elusive (Figure 1).

Sparse data suggest that the role of probiotics may be multifactorial [11]; they have the ability to restore microbial commensal flora populations by eliminating pathogenic bacteria, mediate epithelial barrier permeability, alter visceral hypersensitivity, exert local and systematic anti-inflammatory actions, and regulate gut motility, thus affecting FD symptoms’ severity [4,9]. Intestinal dysbiosis has been linked to the pathogenesis of functional gastrointestinal disorders, namely IBS, while subsequent manipulation of the GI microbiota by probiotics administration has been shown to alleviate symptoms [12]. Nonetheless, the mechanism of action of probiotics in improving IBS symptoms remains unknown, with the ability to modify the expression of gut pain receptors or normalization in the interleukin levels being reported as potential ones [13,14]. Luminal dysbiosis is a phenomenon that seems to be present also in FD, and probiotics can primarily reverse in favor of the commensal flora by directly inhibiting pathogenic bacteria in intestinal epithelial cells, as well as the growth of pathogens; thus, this might be considered as a possible treatment [15]. *Escherichia coli*/*Shigella* are major sources of toxic lipopolysaccharides that can delay gastric emptying, while the ingestion of probiotics, i.e., *Bifidobacterium*, can effectively decrease their levels, restoring small bowel motility function [11]. Similar results were also reported in another trial where *Lactobacillus paracasei* induced a significant reduction in glycogen synthesis and related blood lipids, hence leading to improvement in the gut motility [16]. Emerging data also delineate the pivotal role of probiotics in maintaining the duodenal mucosal integrity. Duodenal barrier defects seem to be a key player in the pathophysiology of FD, with this phenomenon allowing intraluminal antigens of various origin, i.e., food, microbes to exert both a local and systemic immune response, giving birth to dyspeptic symptoms [17]. In this regard, probiotics interfere with the upregulation of genes and expression of proteins involved in tight junctions’ structural component compositions, namely *occludin* and *zonula occludens 1* (ZO-1), while their administration promotes claudin 3 expression, effectively resuming normal function of the impaired intestinal barrier [10,18,19]. Similarly, probiotics have been reported to effectively reduce the altered mucosal permeability by producing metabolites, namely short chain fatty acids (SCFAs) [20,21]. SCFAs are active metabolites produced via the bacterial fermentation of dietary fibers with significant neuroactive properties that contribute to the functional axis between the intestine and central nervous system (CNS). In detail, probiotics can induce SCFA production via the proliferation of beneficial bacteria or complex carbohydrate fermentation; these SCFAs can then affect gut–brain communication through a series of immunological, endocrine, molecular, and vagal pathways. Moreover, SCFAs are involved in interleukin secretion and vagal afferent interactions affecting systemic inflammation, a mechanism that has been documented to contribute in the pathogenesis of FD [22]. Additionally, it has been shown that SCFAs may induce proximal gastric relaxation and alter the intestinal motility through the activation of SCFA receptors, as well as interfere in intestinal secretion through the release of the intestinal hormone peptide YY (PYY) or serotonin from the enterochromaffin cells, the latter not only regulating normal GI transit but also contributing to visceral hypersensitivity and methane-induced IBS [21,23]. Visceral hypersensitivity seems to indeed be an aspect of the FD spectrum pathophysiology, where probiotics might be implicated by regulating pain receptor expression throughout the GI system [24]. Finally, the presence of both local and systemic immune activation has been documented in FD patients [25]. This is considered to be the final step of a complex cascade process, where gut-homing T lymphocytes fail to adequately mediate the inflammatory response to luminal antigens, eventually disrupting the mucosal homeostasis that leads to symptoms occurrence [17]. Probiotics have the potential to interfere with Toll-like Receptors (TLRs—TLR2 and TLR4)—through metabolites production by the gut microbiota—or even regulate proinflammatory cytokines production itself [26].

## 4. The Evidence

The available data from individual studies assessing the efficacy of probiotics in FD are summarized in Table 1.

### 4.1. Data from Cohort Studies

Probiotics and prebiotics are defined as “non digestible food ingredient that beneficially affects the host by selectively stimulating the growth and/or the activity of one or a limited number of bacteria in the colon” [27]. In this aspect, they could be an alternative beneficial therapy for FD, targeting duodenal dysbiosis [4,7]. One of the first studies that evaluated this hypothesis was conducted by Ianiro et al. [28] In this proof-of-concept study, 44 FD—according to the Rome III criteria—outpatients received a mixture of probiotic strains (*Lactobacillus reuterii*, *Lactobacillus rhamnosus*, and *Saccharomyces boulardi*), after thorough medical investigations that excluded organic causes of dyspepsia, as well as other diseases, i.e., *Helicobacter pylori* (*H. pylori*) infection, small intestinal bacterial overgrowth, and lactose malabsorption. After a short treatment period of only 7 days, these patients conveyed significant improvement of dyspeptic symptoms, i.e., nausea and pain/discomfort in the abdominal upper quadrants, as well as gastric distension/postprandial fullness. Shortly after, another small study set out to investigate the structure of gastric microbiota in FD between 44 healthy controls and FD 44 patients [29]. The authors went a step further by treating patients with FD with a yogurt containing a probiotic strain of *Lactobacillus gasseri* OLL2716 for a 12-week-treatment period, aiming to evaluate potential effects on the microbiological parameters and symptoms. Their results indicated that the gastric fluid volume was increased in the FD cohort compared to the controls and subsequently decreased in those FD patients whose symptoms were improved by the probiotic treatment. Moreover, the probiotic treatment reversed the dysbiosis evident in FD patients to the extent that the structure and diversity were almost normal. In detail, their secondary analysis showed that *Prevotella* spp. significantly increased after the probiotic treatment (*p* = 0.001), and more importantly, this stomach-related parameter also had a clinical impact, as it inversely correlated with the severity of PDS symptoms (r = 0.52, *p* = 0.009). In a similar study, Igarashi and colleagues evaluated the gastric microbiota composition in FD patients compared to healthy controls and assessed the impact of probiotics consumption on the microbiota [30]. Twenty-four Japanese patients meeting the Rome III criteria for FD and 21 age/gender-matched healthy volunteers were included, while, in all subjects, the total volume of gastric fluid was sampled after overnight fasting using a nasogastric tube, and the bile acids concentration was evaluated. FD patients received treatment with a probiotic strain of *Lactobacillus gasseri* OLL2716 (LG21), and PCR amplification of the 16S rRNA gene with next-generation sequencing for taxonomic analyses was conducted. Their results support that Bacteroidetes species were in larger populations than Proteobacteria in FD patients compared to the controls (*Bacteroidetes* > *Proteobacteria abundance*), while *Acidobacteria* were absent. Of note, bacterial species that mainly inhabit the intestine, such as *Escherichia*/*Shigella* and *Bifidobacterium longum*, were significantly higher in FD patients than in the control group. From the clinical point of view, probiotic treatment successfully normalized the composition of the gastric microbiota to that found in volunteers. The abundance of *Bacteroidetes* over *Proteobacteria* markedly decreased after treatment, while, contrariwise, both the prevalence and abundance of Acidobacteria increased, with an interindividual variation analysis showing the disappearance of intestinal-type genera after probiotic treatment. Although no clinical endpoints were evaluated, this study provided data for the FD-type phylum profile and demonstrated a clearcut restoration of the FD-type profile to the normal-type profile after probiotic treatment. Trying to provide further clarifications, studies including larger populations and longer treatment periods were designed. Drago et al. [31] administered, in the largest cohort of FD patients (*n* = 2676) so far, a combination of probiotics alone or with prokinetics, antacids, or proton-pump inhibitors for a time period of 30 days.

**Table 1:** Studies assessing the efficacy of probiotics in functional dyspepsia.

Study—Ref	Country	FD Diagnosis	Probiotic (Type/Duration)	Primary Outcome
*Cohort Studies*				
Ianiro et al. [28]	Italy	Rome III	Lactobacillus reuterii 100 billion/g (0.04 g), Lactobacillus rhamnosus GG 350 billion/g (0.1143 g), Saccharomyces boulardi 20 billion/g (0.08 g), vitamin B6 hydrochloride (0.00102 g), inositol (0.025 g), silica (0.01255 g); Q10 coenzyme (25 mg)—7 days	Control in symptoms by using Rome III criteria
Nakae et al. [29]	Japan	Rome III	One hundred and eighteen grams of yogurt containing 109 colony-forming units of Lactobacillus gasseri OLL2716 (LG21) (LG21 yogurt) every day for 12 weeks	Symptoms of GERD (FSSG) questionnaire
Igarashi et al. [30]	Japan	Rome III	One hundred and eighteen grams of yogurt containing 109 colony-forming units (CFUs) of Lactobacillus gasseri OLL2716 (LG21) (LG21 yogurt) every day for 12 weeks	Symptoms of GERD (FSSG) questionnaire
Drago et al. [31]	Italy	Rome IV	Lacticaseibacillus rhamnosus LR04 (DSM 16605), Lactiplantibacillus pentosus LPS01 (DSM 21980), Lactiplantibacillus plantarum LP01 (LMG P-21021), and Lactobacillus delbrueckii subsp. Delbruekii LDD01 (DMS 22106) for 30 days in four groups: (1) probiotics alone, (2) probiotics plus proton-pump inhibitors, (3) probiotics plus prokinetics, and (4) probiotic plus antacid	Presence of specific clinical symptoms associated with PDS (postprandial filling and early satiety) and EPS (epigastric pain and epigastric burning) at study beginning (T0) and study end (T1)
Sun et al. [32]	China	Rome IV	One hundred milliliters (2 bottles/d, 100 mL/bottle) of beverage containing Lactobacillus paracasei LC-37 for 4 weeks	Change in digestive symptom scores at baseline, after 14 and 28 days of treatment
**Randomized Controlled Trials**				
Kim et al. [33]	USA	Rome II	Four grams of probiotics: 50 million CFU (six species): Lactobacillus acidophilus, Bifidobacterium bifidum, Bacillus subtilis, Lactobacillus bulgaricus, Lactobacillus lactis, and Bacillus lichenformis once a day >12 weeks	Gastrointestinal Quality of Life Index (GIQLI) improvement
Ohtsu et al. [34]	Japan	Rome III	Eighty-five grams of yogurt containing Lactobacillus gasseri OLL2716 (L. gasseri OLL2716—amount of L. gasseri OLL2716 per unit of yogurt: 10 9 CFU or higher) for 12 weeks	Global assessment (impression of effect of the 12-week test food intake on gastric symptoms)
Wauters et al. [35]	Belgium	Rome IV	Bacillus coagulans MY01 and Bacillus subtilis MY02, 2·5 × 10^9^ colony-forming units per capsule, taken twice per day with meals for 8 weeks and open-label extension phase of 8 weeks	Proportion of clinical responders; decrease of at least 0·7 in PDS score at week 8 in FD with baseline PDS scores of 1 or more (at least mild symptom scores) on the LPDS diary in the entire cohort (on and off proton-pump inhibitors)

FD: functional dyspepsia; CFU: colony-forming units (as defined in each study); GI: gastrointestinal; NA: not available; FSSG, frequency scale for the symptoms of GERD; PDS: postprandial distress syndrome; LPDS: Leuven Postprandial Distress Scale.

Participants with FD, according to the Rome IV criteria, were called to assess their symptoms using a progressive score scale at enrollment and 15 days post-treatment. Overall, the majority of patients in all four pharmacological therapy arms reported improvement in dyspeptic symptoms after treatment. Perhaps the most interesting finding was the fact that probiotic administration alone was particularly beneficial for PDS patients, resulting in the lowest prevalence of symptoms following treatment (absence of postprandial filling in 14.6% at enrollment and 71.5% post-treatment (*p* < 0.0001) and absence of early satiety in 13.8% at enrollment and 69.5% post-treatment (*p* < 0.0001), respectively). Consistently, the addition of probiotics to any evaluated pharmacological treatment (proton-pump inhibitors, prokinetics, or antacids) also resulted in the resolution of symptoms. As far as patients classified in the EPS subgroup are considered, no definite between-treatment differences were reported. Still, it should be noted that, also in EPS, a combination of probiotics with an established pharmacological therapy, i.e., proton-pump inhibitor, resulted in a statistically significant improvement in both epigastric pain and epigastric burning. These data suggest that different underlying pathological mechanisms may relate to each subset of FD patients, and hence, individualized therapeutic approaches should be applied. Further insight into the clinical outcomes of the probiotic administration in FD patients is provided by a contemporary open study that investigated the effect of a commercially available beverage containing *Lactobacillus paracasei* LC-37 (LC-37) on FD symptoms alleviation [32]. In detail, 26 participants evaluated the severity of their symptoms based on a dedicated questionnaire after 14 and 28 days of treatment with the probiotic beverage, respectively. Of note, a significant amelioration in the symptom’s severity (*p* < 0.05) was recorded at 14 days, while abdominal pain and belching were totally absent after 28 days of treatment. Similar to the aforementioned studies, the authors recorded a significant increase (*p* < 0.05) in SCFA feces concentrations (acetate, propionate, and butyrate) along with alterations in the microbial community composition between the baseline and after 28 days of probiotic administration. At the phylum level, *Firmicutes*, *Bacteroidetes*, *Proteobacteria*, and *Actinobacteria* dominated the fecal microbiota population, while, at the genus level, the probiotic treatment significantly increased the abundance of *Lactobacillus*, *Lactococcus*, and *Weissella* (*p* < 0.05) while decreasing that of *Lachnocliostridium* (*p* < 0.05). the authors went a step further by analyzing fecal nonvolatile metabolites, showing that probiotic use was not only associated with the upregulation of some metabolites, including malonic acid, benzoic acid, pelargonic acid, benzoylformic acid, 1-methylhydantoin, and pipecolic acid, but, at the same time, with the downregulation of others, i.e., carbohydrates, lipids, organic acids, peptides, and steroids. These data support the notion that probiotics may eventually have the potential to shift the tide in favor of beneficial bacterial flora, providing an alternative promising treatment for FD.

### 4.2. Data from Randomized Controlled Trials (RCT)

More than a decade ago, Kim et al. [33], in a well-designed, 12-week randomized, double-blind, placebo-controlled clinical trial of 24 FD patients, evaluated the comparable efficacy of five different combinations of probiotics and nutrients or a placebo. Although the symptoms’ severity as assessed by the Gastrointestinal Quality of Life Index (GIQLI), visual analogue scale (VAS), SF-36, and lactulose and mannitol test (LMT) did not change significantly (*p* > 0.05) after treatment, it should be noted that clinical improvement in all GI symptoms was indeed evident. Thereafter, the results from one Asian and one European center came to light. The former, in a double-blind, parallel group, placebo-controlled, randomized, controlled trial, enrolled 116 (*H. pylori—*negative) FD individuals to ingest a *Lactobacillus gasseri* (OLL2716) containing yogurt or placebo over a 12-week period [34]. At the end of this period, participants were asked to rate a global assessment and the severity of FD-related symptoms according to predesigned questionnaires. The overall effect on gastric symptoms was more favorable in the *Lactobacillus gasseri* OLL2716 group; however, the difference failed to reach statistical significance (*p* = 0.073). Notably, significantly higher elimination rates for the FD symptoms among those receiving the probiotics compared to placebo were recorded (17.3% vs. 35.2%, *p* = 0.048), with this finding being once again evident in the PDS but not in the EPS. This suggests that the therapeutic effect of the probiotic might be focused mainly on gastric motility abnormalities implicated in PDS. Wauters and colleagues—in the first randomized, double-blind, placebo-controlled study evaluating the efficacy of spore-forming probiotics—demonstrated that 8 weeks of treatment with probiotics (*Bacillus coagulans MY01* and *Bacillus subtilis MY02*) resulted in a significantly higher proportion of clinical responders (defined as a decrease of at least 0.7 in the PDS score at week 8) as compared to the placebo (48% vs. 20%; RR 1.95 (95% CI 1.07–4.11); *p* = 0.028) [35]. Moreover, this beneficial effect of probiotics was maintained during the open-label extension phase, highlighting the long-term efficacy of the treatment. Beyond patient-reported outcomes, the authors also evaluated changes in a series of biological parameters, including plasma high-sensitivity C-reactive protein, lipopolysaccharide-binding protein, cytokines, peripheral blood mononuclear cells (PBMCs), and fecal microbiota, in an effort to provide further insights into the complex interplay between the immune system, microbiota, and luminal environment. Although the C-reactive protein or lipopolysaccharide-binding protein values were similar between the two groups in the first 8 weeks, IL-17A significantly decreased. In addition, probiotics use was associated with a significant decrease in Th17 (decreased amounts of T-helper Th-17 cells) and Th2 signaling, as well as gut-homing T cells, suggesting that they are implicated in both local and systemic immune activation pathways. Moreover, these changes were more profound among patients receiving probiotics, along with proton-pump inhibitors (PPIs) that remain, to date, the mainstay of therapy in FD, suggesting additional benefits on chronic alterations with PPIs. Regarding the fecal microbiota analysis, spore-forming probiotics altered α-diversity neither after 8 nor 16 weeks of treatment, with only a proportional but not quantitative increase in *Faecalibacterium* and abundances of *Roseburia* and *Leuconostocaceae* as well. Finally, a significant reduction in the prevalence of small intestinal bacterial overgrowth (SIBO) after 8 weeks of treatment was found (7% vs. 38%; RR 0.26 (0.05–0.96)), suggesting a potential interaction of intestinal dysbiosis with symptom generation in a subgroup of patients with FD, finding that is consistent with the results reported in other studies that specifically addressed this issue [36,37].

### 4.3. Data from Meta-Analysis

The efficacy of probiotics for FD has also been at the focal point of a recently published meta-analysis assessing data exclusively from RCTs [15]. The authors reported that the use of probiotics was not associated with significant improvement in FD symptoms (RR = 1.13; 95% CI: 0.99–1.28; I^2^ = 0%, *p* = 0.67) and also failed to provide any other potentially valuable evidence. Before, however, any conclusion is to be made, some observations call for cautious interpretation of the results. For this meta-analysis, studies with different FD definitions, populations, i.e., *H. pylori*-positive, and outcomes, i.e., addition of a probiotic to increase the efficacy of the *H. Pylori* eradication treatment, were enrolled. Although feasible, any effort to mathematically harmonize data from irrelevant studies increases the risk for heterogeneity and bias. Similarly, the type of strain, dose, and duration of probiotic administration are cofounders that can be effectively addressed only within a meta-regression analysis. In this regard, this work provides not only little evidence but also a questionable quality that can neither support nor reject the hypothesis.

## 5. Critical Appraisal and Future Directions

Several lines of evidence suggest that probiotics could be an appealing future alternative in our therapeutic armamentarium for FD. However, there is still a lot of ground to cover, given the fact that there are several issues related to the quality of the available studies that should be taken into account. First, the definition of FD itself is not uniform among studies. Trials extend over a 15-year period, during which the Rome criteria for FD have undergone substantial amendments and been optimized by the introduction of new therapeutic modalities; hence, changes in the terminology or necessary medical investigations to expedite the diagnosis constitute the populations enrolled in each one probably not comparable and generalizable in other settings. Another matter that should be delineated is the fact that, in most of the studies, the status of an ongoing *H. pylori* infection is not clearly stated. *H. pylori*-related dyspepsia represents a distinct clinical condition that under no circumstance should be confused with FD [5]. In this regard, the Rome IV criteria, as well as the current guidelines, advocate that every patient with dyspeptic symptoms should be tested for *H. pylori* (noninvasively or at gastroscopy), and only those with persistent symptoms 6 to 12 months after successful *H. pylori* eradication should be considered to have FD [2,5,38]. Perhaps the most worrisome issue is the fact that each study evaluated a different probiotic strain or species over a variable time period and at different dosages. Although the use of probiotics may appear apparent in some of the reviewed studies, this significant heterogeneity among the several regimens used prevents from drawing solid conclusions about the potential superiority of any agent and incorporation as a therapeutic approach. To make things even more conflicting, the majority of the studies are not designed to address a definitive causal relationship rather than merely to establish a potential association. Finally, it should be noted that endpoints in studies that are based on patient-reported outcomes always raise concerns about subjectivity, while several caveats, particularly in their design (inadequate statistical power and short follow-up period), are also to be noted. Nonetheless, the current studies show the way for future research. Identification of the heterogeneity of treatment effects in larger studies assessing the impact of probiotics in many different populations could eventually allow us to transcend from empirically administered therapies to specifically orientated, individualized approaches in different populations and FD subtypes.

## 6. Conclusions

While evidence to recommend probiotics use in everyday clinical practice for FD treatment may be currently lacking, physicians should be aware of these therapeutic options as they consider strategies for optimizing FD treatment. This topic is likely to grow in importance as forthcoming studies strengthen this scant evidence for probiotics in different populations and FD subtypes, highlighting interactions between the microbiota and host crosstalk as plausible underlying mechanisms, which will help to establish probiotics as a novel, tailored therapeutic approach for FD.

## Data Availability

All data presented are available within the manuscript.

## Supplementary Materials

The following supporting information can be downloaded at: https://www.mdpi.com/article/10.3390/microorganisms11020351/s1: Table S1: Search Strategy.

## References

[1] Enck P., Azpiroz F., Boeckxstaens G., Elsenbruch S., Feinle-Bisset C., Holtmann G., Lackner J.M., Ronkainen J., Schemann M., Stengel A. (2017). Functional dyspepsia. Nat. Rev. Dis. Prim..

[2] Stanghellini V., Chan F.K., Hasler W.L., Malagelada J.R., Suzuki H., Tack J., Talley N.J. (2016). Gastroduodenal Disorders. Gastroenterology.

[3] Wauters L., Dickman R., Drug V., Mulak A., Serra J., Enck P., Tack J., Group E.F.C., Accarino A., Barbara G. (2021). United European Gastroenterology (UEG) and European Society for Neurogastroenterology and Motility (ESNM) consensus on functional dyspepsia. United Eur. Gastroenterol. J..

[4] Wauters L., Talley N.J., Walker M.M., Tack J., Vanuytsel T. (2020). Novel concepts in the pathophysiology and treatment of functional dyspepsia. Gut.

[5] Moayyedi P., Lacy B.E., Andrews C.N., Enns R.A., Howden C.W., Vakil N. (2017). ACG and CAG Clinical Guideline: Management of Dyspepsia. Am. J. Gastroenterol..

[6] Tack J., Carbone F. (2017). Functional dyspepsia and gastroparesis. Curr. Opin. Gastroenterol..

[7] Tziatzios G., Gkolfakis P., Papanikolaou I.S., Mathur R., Pimentel M., Giamarellos-Bourboulis E.J., Triantafyllou K. (2020). Gut Microbiota Dysbiosis in Functional Dyspepsia. Microorganisms.

[8] Talley N.J. (2020). What Causes Functional Gastrointestinal Disorders? A Proposed Disease Model. Am. J. Gastroenterol..

[9] Ford A.C., Quigley E.M., Lacy B.E., Lembo A.J., Saito Y.A., Schiller L.R., Soffer E.E., Spiegel B.M., Moayyedi P. (2014). Efficacy of prebiotics, probiotics, and synbiotics in irritable bowel syndrome and chronic idiopathic constipation: Systematic review and meta-analysis. Am. J. Gastroenterol..

[10] Ukena S.N., Singh A., Dringenberg U., Engelhardt R., Seidler U., Hansen W., Bleich A., Bruder D., Franzke A., Rogler G. (2007). Probiotic Escherichia coli Nissle 1917 inhibits leaky gut by enhancing mucosal integrity. PLoS ONE.

[11] Hosseini A., Nikfar S., Abdollahi M. (2012). Probiotics use to treat irritable bowel syndrome. Expert Opin. Biol. Ther..

[12] Pimentel M., Lembo A. (2020). Microbiome and Its Role in Irritable Bowel Syndrome. Dig. Dis. Sci..

[13] O’Mahony L., McCarthy J., Kelly P., Hurley G., Luo F., Chen K., O’Sullivan G.C., Kiely B., Collins J.K., Shanahan F. (2005). Lactobacillus and bifidobacterium in irritable bowel syndrome: Symptom responses and relationship to cytokine profiles. Gastroenterology.

[14] Rousseaux C., Thuru X., Gelot A., Barnich N., Neut C., Dubuquoy L., Dubuquoy C., Merour E., Geboes K., Chamaillard M. (2007). Lactobacillus acidophilus modulates intestinal pain and induces opioid and cannabinoid receptors. Nat. Med..

[15] Zhang J., Wu H.M., Wang X., Xie J., Li X., Ma J., Wang F., Tang X. (2020). Efficacy of prebiotics and probiotics for functional dyspepsia: A systematic review and meta-analysis. Medicine.

[16] Gudina E.J., Teixeira J.A., Rodrigues L.R. (2010). Isolation and functional characterization of a biosurfactant produced by Lactobacillus paracasei. Colloids Surf. B Biointerfaces.

[17] Vanheel H., Vicario M., Vanuytsel T., Van Oudenhove L., Martinez C., Keita A.V., Pardon N., Santos J., Soderholm J.D., Tack J. (2014). Impaired duodenal mucosal integrity and low-grade inflammation in functional dyspepsia. Gut.

[18] Patel R.M., Myers L.S., Kurundkar A.R., Maheshwari A., Nusrat A., Lin P.W. (2012). Probiotic bacteria induce maturation of intestinal claudin 3 expression and barrier function. Am. J. Pathol..

[19] Caviglia G.P., Rosso C., Ribaldone D.G., Dughera F., Fagoonee S., Astegiano M., Pellicano R. (2019). Physiopathology of intestinal barrier and the role of zonulin. Minerva Biotecnol..

[20] Kaji I., Iwanaga T., Watanabe M., Guth P.H., Engel E., Kaunitz J.D., Akiba Y. (2015). SCFA transport in rat duodenum. Am. J. Physiol. Gastrointest. Liver Physiol..

[21] Camilleri M. (2012). Peripheral mechanisms in irritable bowel syndrome. N. Engl. J. Med..

[22] Dalile B., Van Oudenhove L., Vervliet B., Verbeke K. (2019). The role of short-chain fatty acids in microbiota-gut-brain communication. Nat. Rev. Gastroenterol. Hepatol..

[23] Yano J.M., Yu K., Donaldson G.P., Shastri G.G., Ann P., Ma L., Nagler C.R., Ismagilov R.F., Mazmanian S.K., Hsiao E.Y. (2015). Indigenous bacteria from the gut microbiota regulate host serotonin biosynthesis. Cell.

[24] Simren M., Tornblom H., Palsson O.S., van Tilburg M.A.L., Van Oudenhove L., Tack J., Whitehead W.E. (2018). Visceral hypersensitivity is associated with GI symptom severity in functional GI disorders: Consistent findings from five different patient cohorts. Gut.

[25] Burns G., Carroll G., Mathe A., Horvat J., Foster P., Walker M.M., Talley N.J., Keely S. (2019). Evidence for Local and Systemic Immune Activation in Functional Dyspepsia and the Irritable Bowel Syndrome: A Systematic Review. Am. J. Gastroenterol..

[26] Anitha M., Vijay-Kumar M., Sitaraman S.V., Gewirtz A.T., Srinivasan S. (2012). Gut microbial products regulate murine gastrointestinal motility via Toll-like receptor 4 signaling. Gastroenterology.

[27] Wu R.Y., Maattanen P., Napper S., Scruten E., Li B., Koike Y., Johnson-Henry K.C., Pierro A., Rossi L., Botts S.R. (2017). Non-digestible oligosaccharides directly regulate host kinome to modulate host inflammatory responses without alterations in the gut microbiota. Microbiome.

[28] Ianiro G., Pizzoferrato M., Franceschi F., Tarullo A., Luisi T., Gasbarrini G. (2013). Effect of an extra-virgin olive oil enriched with probiotics or antioxidants on functional dyspepsia: A pilot study. Eur. Rev. Med. Pharmacol. Sci..

[29] Nakae H., Tsuda A., Matsuoka T., Mine T., Koga Y. (2016). Gastric microbiota in the functional dyspepsia patients treated with probiotic yogurt. BMJ Open Gastroenterol..

[30] Igarashi M., Nakae H., Matsuoka T., Takahashi S., Hisada T., Tomita J., Koga Y. (2017). Alteration in the gastric microbiota and its restoration by probiotics in patients with functional dyspepsia. BMJ Open Gastroenterol..

[31] Drago L., Meroni G., Pistone D., Pasquale L., Milazzo G., Monica F., Aragona S., Ficano L., Vassallo R., Gastrobiota G. (2021). Evaluation of main functional dyspepsia symptoms after probiotic administration in patients receiving conventional pharmacological therapies. J. Int. Med. Res..

[32] Sun E., Zhang X., Zhao Y., Li J., Sun J., Mu Z., Wang R. (2021). Beverages containing Lactobacillus paracasei LC-37 improved functional dyspepsia through regulation of the intestinal microbiota and their metabolites. J. Dairy Sci..

[33] Kim S.L., Hilli L., Orlowski J., Kupperman J.L., Baral M., Waters R.F. (2006). Efficacy of probiotics and nutrients in functional gastrointestinal disorders: A preliminary clinical trial. Dig. Dis. Sci..

[34] Ohtsu T., Takagi A., Uemura N., Inoue K., Sekino H., Kawashima A., Uchida M., Koga Y. (2017). The Ameliorating Effect of Lactobacillus gasseri OLL2716 on Functional Dyspepsia in Helicobacter pylori-Uninfected Individuals: A Randomized Controlled Study. Digestion.

[35] Wauters L., Slaets H., De Paepe K., Ceulemans M., Wetzels S., Geboers K., Toth J., Thys W., Dybajlo R., Walgraeve D. (2021). Efficacy and safety of spore-forming probiotics in the treatment of functional dyspepsia: A pilot randomised, double-blind, placebo-controlled trial. Lancet Gastroenterol. Hepatol..

[36] Tziatzios G., Giamarellos-Bourboulis E.J., Papanikolaou I.S., Pimentel M., Dimitriadis G.D., Triantafyllou K. (2017). Is small intestinal bacterial overgrowth involved in the pathogenesis of functional dyspepsia?. Med. Hypotheses.

[37] Tziatzios G., Gkolfakis P., Papanikolaou I.S., Mathur R., Pimentel M., Damoraki G., Giamarellos-Bourboulis E.J., Dimitriadis G., Triantafyllou K. (2020). High prevalence of small intestinal bacterial overgrowth among functional dyspepsia patients. Dig. Dis..

[38] Sugano K., Tack J., Kuipers E.J., Graham D.Y., El-Omar E.M., Miura S., Haruma K., Asaka M., Uemura N., Malfertheiner P. (2015). Kyoto global consensus report on Helicobacter pylori gastritis. Gut.

