# Supplementary material for: Probiotics in Functional Dyspepsia
Source: Microorganisms. 2023 Jan 31;11(2):351. doi: 10.3390/microorganisms11020351 (PMC9964889; doi:10.3390/microorganisms11020351)
Supplement: Supplementary file 1 [file microorganisms-11-00351-s001.zip › microorganisms-2133123-supplementary.pdf]

Supplementary File Table S1. Search Strategy

PubMed (Date Run: 14/11/2022)

Step	Search strategy	Found	Time
**#1**	Search: functional dyspepsia Sort by: Publication Date ("functional"[All Fields] OR "functional s"[All Fields] OR "functionalities"[All Fields] OR "functionality"[All Fields] OR "functionalization"[All Fields] OR "functionalizations"[All Fields] OR "functionalize"[All Fields] OR "functionalized"[All Fields] OR "functionalizes"[All Fields] OR "functionalizing"[All Fields] OR "functionally"[All Fields] OR "functionals"[All Fields] OR "functioned"[All Fields] OR "functioning"[All Fields] OR "functionings"[All Fields] OR "functions"[All Fields] OR "physiology"[MeSH Subheading] OR "physiology"[All Fields] OR "function"[All Fields] OR "physiology"[MeSH Terms]) AND ("dyspepsia"[MeSH Terms] OR "dyspepsia"[All Fields] OR "dyspepsias"[All Fields]) Translations functional: "functional"[All Fields] OR "functional's"[All Fields] OR "functionalities"[All Fields] OR "functionality"[All Fields] OR "functionalization"[All Fields] OR "functionalizations"[All Fields] OR "functionalize"[All Fields] OR "functionalized"[All Fields] OR "functionalizes"[All Fields] OR "functionalizing"[All Fields] OR "functionally"[All Fields] OR "functionals"[All Fields] OR "functioned"[All Fields] OR "functioning"[All Fields] OR "functionings"[All Fields] OR "functions"[All Fields] OR "physiology"[Subheading] OR "physiology"[All Fields] OR "function"[All Fields] OR "physiology"[MeSH Terms] dyspepsia: "dyspepsia"[MeSH Terms] OR "dyspepsia"[All Fields] OR "dyspepsias"[All Fields]	7889	15:25:27
**#2**	Search: probiotics Sort by: Publication Date "probiotic s"[All Fields] OR "probiotical"[All Fields] OR "probiotics"[MeSH Terms] OR "probiotics"[All Fields] OR "probiotic"[All Fields] Translations probiotics: "probiotic's"[All Fields] OR "probiotical"[All Fields] OR "probiotics"[MeSH Terms] OR "probiotics"[All Fields] OR "probiotic"[All Fields]	40270	15:27:23
**#3**	#1 AND #2	91	15:28:54
